# Function and Regulation of Clustered Regularly Interspaced Short Palindromic Repeats (CRISPR) / CRISPR Associated (Cas) Systems

**DOI:** 10.3390/v4102291

**Published:** 2012-10-19

**Authors:** Corinna Richter, James T. Chang, Peter C. Fineran

**Affiliations:** Department of Microbiology and Immunology, University of Otago, PO Box 56, Dunedin 9054, New Zealand; Email: ricco896@student.otago.ac.nz (C.R.); james.chang@otago.ac.nz (J.T.C.)

**Keywords:** phages, plasmids, horizontal gene transfer, CRISPR, Cas, cascade, PAM, crRNA, resistance

## Abstract

Phages are the most abundant biological entities on earth and pose a constant challenge to their bacterial hosts. Thus, bacteria have evolved numerous ‘innate’ mechanisms of defense against phage, such as abortive infection or restriction/modification systems. In contrast, the **c**lustered **r**egularly **i**nterspaced **s**hort **p**alindromic **r**epeats (CRISPR) systems provide acquired, yet heritable, sequence-specific ‘adaptive’ immunity against phage and other horizontally-acquired elements, such as plasmids. Resistance is acquired following viral infection or plasmid uptake when a short sequence of the foreign genome is added to the CRISPR array. CRISPRs are then transcribed and processed, generally by **C**RISPR **as**sociated (Cas) proteins, into short interfering RNAs (crRNAs), which form part of a ribonucleoprotein complex. This complex guides the crRNA to the complementary invading nucleic acid and targets this for degradation. Recently, there have been rapid advances in our understanding of CRISPR/Cas systems. In this review, we will present the current model(s) of the molecular events involved in both the acquisition of immunity and interference stages and will also address recent progress in our knowledge of the regulation of CRISPR/Cas systems.

## 1. Introduction

Bacteria and Archaea are frequently exposed to stresses such as infection from bacteriophages (phage) and other genetic elements. These events can result in horizontal gene transfer (HGT), which is mediated by transduction, transformation or by conjugation of mobile elements such as plasmids [1,2]. HGT can be beneficial for adaptation and survival by rapidly promoting the transfer of genes encoding antibiotic resistance, virulence factors, or the ability to degrade toxic compounds [1]. However, in the absence of any selective advantage, foreign genetic elements can have a fitness cost. This is most evident when prokaryotes are infected by virulent phages. The sheer scale of viral abundance (~10^31^), diversity and rates of infection (~10^25^/s) [3,4] has resulted in the evolution of prokaryotic defenses to maintain the balance in this arms race [5]. 

Bacteria possess multiple methods to regulate genetic flux and resist phage infection. These mechanisms include the mutation or masking of cell surface receptors, restriction-modification, abortive infection and the **c**lustered **r**egularly **i**nterspaced **s**hort **p**alindromic **r**epeats (CRISPR) systems [5,6]. CRISPR systems are a widespread mechanism that equips bacteria with a sequence-specific heritable ‘adaptive immune system’ that has a genetic memory of past genetic incursions (for recent reviews see [7,8,9]).

**Figure 1:**
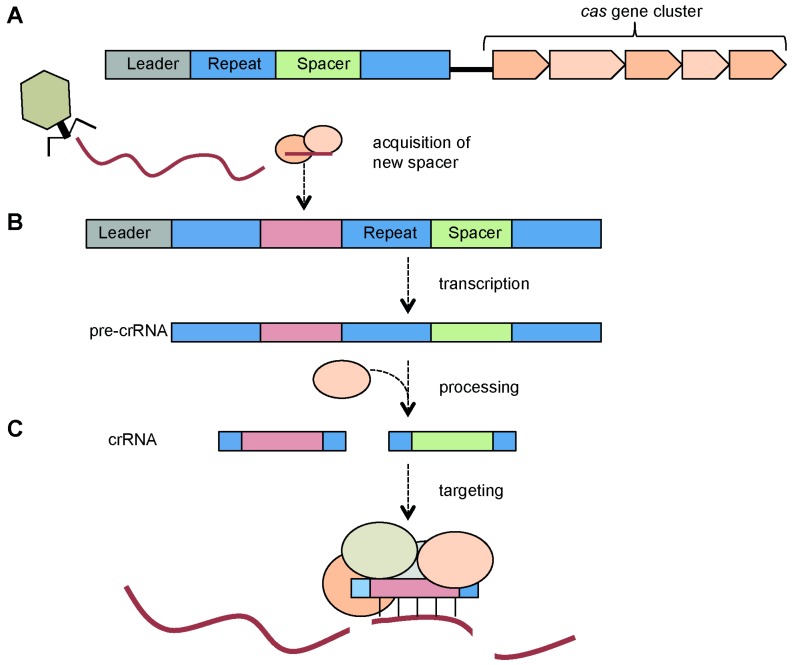
**Overview of** **c**lustered **r**egularly **i**nterspaced **s**hort **p**alindromic **r**epeats (**CRISPR)/C**RISPR **as**sociated (**Cas) adaptive immunity.** (**a**) Adaptation. The CRISPR arrays are composed of short repeats and intervening sequences derived from foreign invaders. Upon infection with a foreign element (e.g., phages or plasmids), part of the genome is typically incorporated into the leader end of the CRISPR array and the repeat is duplicated. The CRISPR arrays are located adjacent to a cluster of *cas* genes; (**b**) crRNA generation. The CRISPRs are transcribed into pre-crRNAs that are then processed into mature crRNAs; (**c**) Interference. The crRNA, in a complex with Cas proteins, binds and degrades the target nucleic acid of the invading element.

CRISPRs use small non-coding RNAs for defense and function in conjunction with **C**RISPR **as**sociated (Cas) proteins. The mechanism of CRISPR/Cas interference involves three phases (Figure 1). Firstly, resistance is acquired via the integration of short sequences from foreign genetic elements (termed spacers) into repetitive genetic elements known as CRISPR arrays. Secondly, CRISPR arrays are then transcribed and processed into small RNAs (crRNAs) by Cas proteins. In the third and final step, targeting of the invading phage or plasmid is mediated by a Cas protein complex that contains crRNAs. During this stage, the crRNA-Cas protein complex then interferes, in a sequence-specific manner, with the foreign nucleic acids. 

Despite sharing mechanistic similarities, there is significant diversity amongst CRISPR/Cas systems. There are three major CRISPR/Cas system types (I-III), which are characterized by a signature protein [10]. The main types are further divided into subtypes (e.g., I-A to I-F) based mainly on the presence of a subtype-specific set of Cas proteins and for some types the repeat sequence of the associated CRISPR array is also taken into account. The last five years have seen rapid advances in our mechanistic/molecular understanding of CRISPR/Cas systems. In this review, we provide an historical context, highlight the current models for the adaptation, processing and interference stages and finally, discuss the regulation of the CRISPR/Cas systems.

## 2. Discovery of CRISPR/Cas Systems

The first report of interspaced palindromic repeat sequences was made by Ishino *et al*. who detected five 29 bp repeats with 32 bp spacers near the *iap* gene of *Escherichia coli* [11]. Further reports were made for *Mycobacterium tuberculosis* [12], *Haloferax* spp. [13] and *Archaeoglobus fulgidus* [14,15]; however, they were not detected in eukaryotic or virus sequences [15]. Mojica and co-workers performed a comparative *in silico* study of those repetitive elements to determine structure and sequence similarity, as well as their phylogenetic distribution [16] showing that CRISPRs display a high degree of homology between phylogenetically distant species and a wide distribution in bacteria and archaea. To date CRISPR/Cas systems have been found in almost 50% of bacterial and 85% of archaeal genome sequences available [17].

Terminology for these repeated elements was inconsistent and included interspaced **s**hort **s**equence **r**epeats (SSR) [18], **sp**acer **i**nterspersed **d**irect **r**epeats (SPIDR) [15] or **s**hort **r**egularly **s**paced **r**epeats (SRSRs) [16]. For simplicity, the name CRISPR (**c**lustered **r**egularly **i**nterspaced **s**hort **p**alindromic **r**epeats) was accepted since it reflects the most important characteristics [19]. 

An important step towards the understanding of the role of CRISPRs was the identification of four genes closely located to the CRISPR arrays [19], which were termed **C**RISPR-**as**sociated (*cas*) genes 1-4. Cas1-4 were only present in genomes containing CRISPR arrays and their predicted functions included helicase and nuclease activities, which led to the hypothesis that CRISPR/Cas systems might be involved in DNA repair [20].

A critical discovery came in 2005 with three independent studies showing that spacers matched sequences of extrachromosomal origin, including phages, prophages and plasmids [21,22,23]. Furthermore, there was a positive correlation between the presence of spacers matching a particular phage and phage resistance [22,23]. In combination with detailed bioinformatics analyses of the Cas proteins, it was hypothesized that CRISPR/Cas systems provide an RNAi-like mechanism of resistance against invading genetic elements [21,22,23,24,25].

The first direct evidence that CRISPR/Cas could protect bacteria from phages or plasmids was provided by two key studies. Firstly, Barrangou *et al*. obtained phage resistant mutants following phage challenge of *Streptococcus thermophilus*. The phage resistant strains had incorporated new spacers matching to the viral genome while removal or addition of spacers matching to the phages resulted in phage sensitivity or resistance, respectively [26]. Subsequently, Marraffini and Sontheimer showed that CRISPR/Cas systems can also prevent both conjugation and transformation of plasmids in *Staphylococcus epidermidis *[27]. Recently, it was shown that the *Streptococcus pneumoniae* CRISPR/Cas can prevent natural transformation. Non-capsulated *S. pneumoniae* with CRISPRs programmed to target capsule genes were unable to be transformed into capsulated strains during infection in mice [28].

## 3. Components of CRISPR/Cas Systems

CRISPR/Cas systems comprise the following critical elements: a CRISPR array, an upstream leader sequence and the *cas* genes (Figure 1A) that are discussed below [19,29]. 

### 3.1. CRISPR Arrays: Repeats, Spacers and Leader Sequence

In the CRISPR array, repeats alternate with spacer sequences [11,30,31,32]. Within arrays, the repeats are typically identical in terms of length and sequence, but small differences do occur [16,29]. In particular, the repeat at the end of an array is often truncated or deviates more from the consensus sequence [17,33]. Repeats are usually between 23 and 47 bp in length and those of the same subtype share a consensus sequence in type I-C, I-E, I-F, and II [10,34,35]. Many repeat sequences show palindromes or short inverted repeats [16] and a predicted stable secondary structure in the form of a stem-loop [34]. However, there are repeats, which are not palindromic and are predicted to be unstructured. This difference in the ability to form secondary structures also has implications for the mechanism of pre-crRNA processing, which will be discussed later [10,34].

In contrast, the spacer sequences are mostly unique within a genome. Many spacers have been matched to sequences originating from extra-chromosomal sources such as phage or plasmids and other transferable elements [21,22,23,36]. Indeed, it is the spacers that confer the sequence-specific immunity against those extra-chromosomal agents [26,33,37]. The sequences in the foreign genome from which spacers are derived are termed protospacers. It is likely that the majority of spacers are phage- or plasmid-derived, but the limited depth of sequence data on these abundant and diverse elements leads to an underestimation. Interestingly, some CRISPR/Cas systems also possess spacers that match sequences elsewhere within their own genome. Their function, if any, is not resolved, but it has been proposed that they have been accidentally incorporated [38]. Spacer length can vary slightly throughout an array (typically by 1–2 nt) and spacer lengths up to 72 bp have been reported, but usually the size is similar to that of the repeats in the same array [17]. 

The third component of the CRISPR array is the leader sequence, which is located upstream of the first repeat [15]. This AT-rich sequence is about 200–500 bp long and includes the promoter necessary for transcription of the array (see below) [39,40,41,42]. The leader region is also important for the acquisition of new spacers [43].

A single genome can harbor more than one CRISPR array. Those can vary considerably in size with the largest identified to date, in *Haliangium ochraceum* DSM 14365, containing 587 repeats [35]. The length of CRISPR arrays appears to correlate with the degree of activity in spacer acquisition, with longer arrays being more active than short arrays and those with degenerate repeat sequences [33]. 

### 3.2. Cas Proteins

In close proximity to the CRISPR array are genes that encode the **C**RISPR **as**sociated (Cas) proteins (Figure 1A) [19,20,24,25,44]. Cas proteins provide the enzymatic machinery required for the acquisition of new spacers from, and targeting, invading elements.

CRISPR/Cas systems are currently classified into type I, II and III, based on the phylogeny and presence of particular Cas proteins [10]. There is further division within each type into subtypes (e.g., type I is composed of type I-A to I-F). The Cas proteins are important for the differentiation between both the major CRISPR/Cas types and the subtypes. Two main groups of Cas proteins can be distinguished. The first group, which includes the core Cas proteins, is found across multiple types or subtypes. Cas1 and Cas2 (Cas2 is sometimes fused to another Cas protein) are found in every Cas operon across the three main types, and Cas1 is considered the universal marker of CRISPR/Cas systems [10,24,25,44]. Cas3, Cas9, and Cas10 are each specific for one major type, serving as signature proteins for type I, II, and III, respectively [10].

The second major group consists of proteins only found within the gene clusters of one particular subtype [10,25]. For example, the Cse and Csy proteins are subtype-specific for type I-E and type I-F systems, respectively. The subtype specific proteins of some systems form a complex involved in targeting and interference, which in the type I-E system is referred to as Cascade (CRISPR-associated complex for antiviral defense) [45,46,47,48,49]. These complexes will be discussed in detail below. The systems can be complex since some bacteria contain multiple CRISPR/Cas subtypes, each of which can have multiple CRISPR arrays that function with the appropriate Cas cluster [18]. In summary, the *cas* cluster for each CRISPR/Cas subtype contains genes encoding Cas1 and Cas2, the signature protein defining the major type and a set of subtype specific proteins. Interestingly, the total number of proteins differs between the types of system, even though the functional principles are similar in the cases studied thus far. It is possible that unexpected functions may reside within Cas proteins that are yet to be characterized. 

## 4. CRISPR/Cas Mechanism

As highlighted in Figure 1, the mechanism of CRISPR/Cas defense involves three stages. Resistance must first be acquired by integrating spacers into the CRISPR arrays. These arrays are then transcribed and processed into short crRNAs. Finally, a crRNA-Cas ribonucleoprotein complex targets the invading nucleic acid for degradation. In the following sections we outline the processes of spacer acquisition, crRNA biogenesis and interference. 

### 4.1. Adaptation via Spacer Acquisition

**Figure 2:**
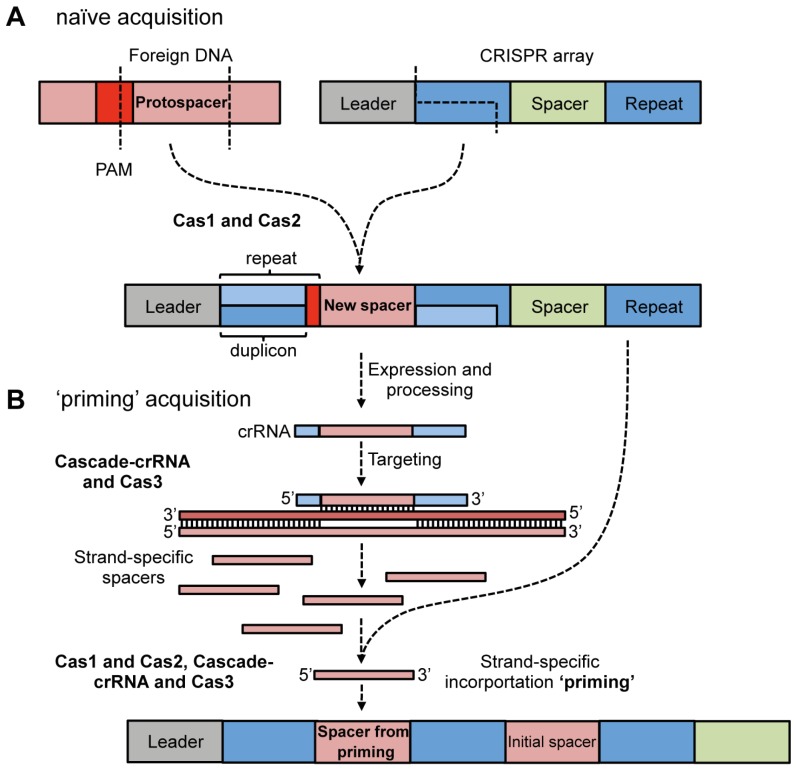
**Model of CRISPR/Cas adaptation **(based on type I-E systems). (**a**) Naïve acquisition. Cas1 and Cas2 are required for acquisition of new spacers. The first repeat at the leader end of the CRISPR array is duplicated and incorporates a new spacer sequence from a protospacer. The final nucleotide of the repeat is not duplicated but is provided by the PAM nucleotide immediately adjacent to the protospacer; (**b**) Priming acquisition. Expression of the CRISPR array and generation of the crRNAs against the foreign DNA results in binding / targeting, which is hypothesized to aid in the generation of precursors for integration (possibly single- or double-stranded). Productive interference is not required for priming. Priming acquisition requires Cascade-crRNA, Cas3 and Cas1 and Cas2 and results in new spacers derived from the same strand as the initial spacer.

The least characterised stage of CRISPR/Cas defense is adaptation, whereby new spacers are acquired from phages and plasmids and, with duplication of the repeat, are added to the leader proximal end of the CRISPR array (Figure 2). A number of metagenomic studies of phage-bacterial dynamics in environmental [50,51] and human [52] niches have provided clear evidence that CRISPR/Cas systems actively acquire new spacers from mobile genetic elements over short time-scales. The first demonstration of spacer incorporation from phages and plasmids in the laboratory was in the type II-A system of *S. thermophilus* [26,53]. Very recently, studies have observed spacer acquisition in the *E. coli* type I-E [43,54,55], *P. aeruginosa* type I-F [56], *Streptococcus agalactiae* type II-A [57] and *Sulfolobus solfataricus* type I and III-B [58] systems. The most well characterized system is the *E. coli* type I-E, so this will be the focus of the following discussion.

#### 4.1.1. Cas Proteins Required for Adaptation

In the *E. coli* type I-E system, Cas1 and Cas2 are required for spacer incorporation from plasmids and phages [43,55]. These recent results are consistent with the dispensable role of Cas1 and Cas2 in crRNA biogenesis / targeting in type I-E [45], type II-A [59] and type III-A [60] systems. The type I-E data is of broad relevance because Cas1 and Cas2 are conserved across all CRISPR/Cas types [10,24]. It is interesting that an insertion mutation of *csn2* inhibited spacer acquisition in the *S. thermophilus* type II-A system, suggesting that, in addition to Cas1 and Cas2, other Cas proteins might play a role [26].

Exactly how Cas1 and Cas2 function is unclear, but biochemical and structural studies offer some clues [61,62,63]. Cas1 is a metal-dependent endonuclease and the *Pseudomonas* Cas1 cleaves dsDNA, generating ~80 bp fragments [61]. The 80 bp dsDNA fragments generated from Cas1 are larger than expected for integration (~32–33 nt), indicating that other factors are involved. Cas1 dimers contain a stirrup-like structure and a positively charged surface that together might be involved in binding dsDNA [9]. Cas2 from *S. solfataricus* cleaves ssRNA at U-rich regions *in vitro* [64], but ssRNA or ssDNA binding or cleavage was not detected for *Desulfovibrio vulgaris* Cas2 [65]. Recently, the *Bacillus halodurans* Cas2 dimer was shown to contain Mg^2+^-dependent endonuclease activity against dsDNA and generated 120 bp products [66]. Despite these studies the exact mechanistic roles of Cas1 and Cas2 in acquisition are unknown. 

There is increasing evidence that some Cas1 and Cas2 proteins may function together directly (e.g., via protein-protein interactions). For example, the type I-A system from *Thermoproteus tenax* contains a Cas1-Cas2 fusion protein, which interacts with Csa4 and Csa1, in what the authors name Cascis (**C**RISPR **as**sociated **c**omplex for the **i**ntegration of **s**pacers) [67]. It is noteworthy that the type I-F Cas3 is a Cas2-Cas3 hybrid, containing an N-terminal Cas2-like domain fused to Cas3 [10,24]. 

#### 4.1.2. Protospacer Selection and Incorporation into CRISPR Arrays

The minimal CRISPR requirements for spacer acquisition in the type I-E system are a single ‘repeat’ and 60 bp of upstream sequence [43]. As the CRISPR promoter is not contained within this 60 bp, this suggests that CRISPR transcription is not required for incorporation, but indicates this region might be recognized by Cas1 and/or Cas2 to enable the directionality of integration at the leader end of the CRISPR array.

In type I-E arrays with multiple repeats, the leader proximal repeat is the one that is duplicated (Figure 2A) [43]. In addition, the protospacer added to the CRISPR array contains the last nt of the PAM motif, which becomes the final nt of the 5' repeat (Figure 2A) [54,55,68]. Therefore, only the first 28 nt of the repeat are duplicated and thus, this unit has been called the ‘duplicon’ [68] and might provide fidelity to the direction of incorporation [69]. This duplicon mechanism cannot apply to all CRISPR/Cas types since the last nucleotide of some PAMs (e.g., CCN in *S. solfataricus*) is not conserved, so could not contribute to the conserved repeats [58]. Interestingly, internal spacer acquisition was recently observed for one CRISPR array in *S. solfataricus* [58], which challenges the dogma that spacer acquisition is always leader proximal [69].

One critical question is how do the CRISPR/Cas systems acquire foreign DNA and not DNA from their own chromosome? In *S. thermophilus*, examination of the protospacers in the phage genomes enabled the identification of a short 3' protospacer adjacent motif (PAM) [37]. PAM sequences have since been identified via bioinformatics for other CRISPR/Cas systems [70]. In *E. coli*, spacer selection was shown to require the presence of a PAM [43], but the short length of PAMs cannot provide the level of discrimination to avoid sampling chromosomal DNA. A self / non-self mechanism does occur, since the incorporation of spacers from the chromosome is rare [43]. It is possible that DNA modification, such as by a restriction-modification-like mechanism, might play a role.

#### 4.1.3. Priming Acquisition

In the type I-E system there is now evidence that acquisition is divided into two stages; (1) naïve- and (2) priming-based acquisition [54,55]. As discussed above naïve acquisition is the initial spacer incorporation from a new foreign element. During priming, the initial spacer ‘primes’ the array for the addition of multiple invader-derived spacers from the same DNA strand as the initial spacer (Figure 2B) [54,55]. Cas1, Cas2, Cascade, Cas3 and a crRNA that matches the invading element are required for priming [55]. The nuclease activity of Cas3 was proposed to be required for the feedback loop, generating precursors for incorporation following the initial targeting event [54]. However, spacers that match the foreign DNA but do not support targeting, due to single nucleotide mutations, still promote priming [55]. Priming is proposed to allow adaptation to phage and plasmids that mutate and escape the initial spacers [55] and multiple spacers would increase resistance and decrease the frequency of escape.

In summary, details of CRISPR adaptation are beginning to come to light, but there are still many questions regarding the exact mechanisms of protospacer recognition, spacer generation, repeat duplication, integration of the new spacer and the roles of the Cas proteins.

### 4.2. Expression, crRNA Generation and Interference

**Figure 3:**
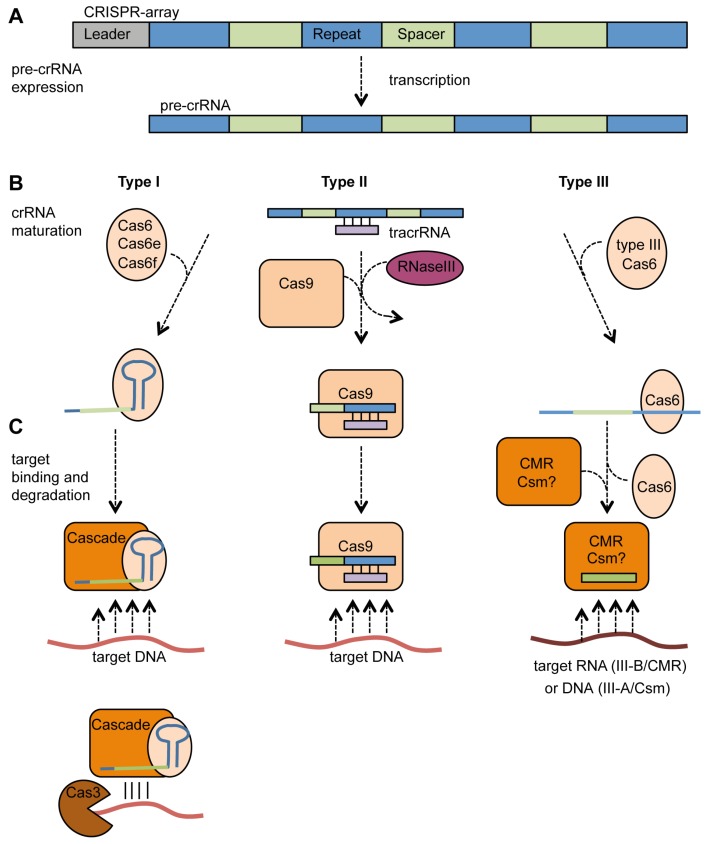
**crRNA generation and target interference in type I, II and III CRISPR/Cas systems**.(**a**) Transcription of the CRISPR array into a pre-crRNA; (**b**) Processing of the pre-crRNA into mature short crRNAs. In type I, RNA cleavage is performed by Cas6-homologues, which bind the repeat stem-loop and stay associated for Cascade formation. In type II, tracrRNA is required for binding and processing of the pre-crRNA by Cas9 and RNaseIII. In type III, Cas6 binds to non-structured repeats and processes the pre-crRNA into crRNA and then dissociates; (**c**) Target binding and cleavage. Type I Cascade binds the DNA target before recruiting Cas3 for degradation. In type II, Cas9 stays associated with the tracrRNA:crRNA complex after processing and subsequently binds and cleaves target DNA. The type III-B CMR-complex binds spacer sequence and targets RNA. It is hypothesized that a type III-A Csm complex forms and this system targets DNA.

Following the acquisition of spacers, the CRISPR array is expressed as a long pre-crRNA that is processed into crRNAs. The mature crRNAs form part of a ribonucleoprotein complex, which targets and degrades the foreign genetic material. As will be outlined below, these processes are similar, yet differ between type I, II, and III systems (Figure 3).

#### 4.2.1. Type I Systems

In type I CRISPR/Cas systems, regions within each repeat form a stem-loop, which is bound by the processing endoribonuclease. The endoribonucleases in type I systems belong to the group of Cas6 proteins. Characterized members of this group include Cas6b in type I-B [71], Cas5d in type I-C [49], Cas6e (CasE, Cse3) in type I-E [45,72,73] and Cas6f (Csy4) in type I-F [42,74]. The pre-crRNA is cleaved in a sequence-specific manner at the downstream base of the stem-loop, producing the mature crRNA consisting of a short 5' repeat handle followed by the spacer sequence and the stem-loop of the next repeat (Figure 3B) [49,71,74,75]. The crRNA and the endoribonuclease stay associated, and might serve as nucleation point [48,49,76] for the formation of Cascade (Figure 3C). Cascade is a ribonucleoprotein complex formed by the subtype-specific Cas proteins and the crRNA, initially identified in type I-E, but later also found in type I-C and I-F [9,45,46,48,49].

The type I-E Cascade has a shape referred to as seahorse-like and has a hexameric backbone formed by Cas7 (CasC, Cse4). The stem-loop of the crRNA is bound to the endoribonuclease Cas6e, which forms the “head” of the seahorse. Cse1 (CasA) forms the “tail” and binds the 3' end of the crRNA at the other end. Cse1 is bridged to Cas6e by a Cse2 (CasB) dimer, and linked by Cas5 (CasD) to the sixth Cas7 protein of the backbone [46,47]. The I-F and I-C complexes share common features with the I-E Cascade in terms of their shape, but also show variation, which is mainly due to different proteins involved. Different numbers of subtype-specific proteins constitute Cascade, three in the type I-C, four in I-F systems, while five distinct proteins are present in the type I-E Cascade [10]. Additional variation occurs at the level of the stoichiometry of individual protein subunits in different subtype Cascade complexes. When three or four proteins are used, the endoribonuclease, with the crRNA bound via the stem-loop, is located at one end of the complex. A homo-multimer of six proteins (Csy3 and Csd2 for I-F, and I-C, respectively) forms the neck or backbone of the complex, which holds the spacer and ensures it is extended for optimal presentation to invading nucleic acids. The end of the complex is formed by a hetero-dimer [48,49]. A type I-A complex from *S. solfataricus* (termed aCASCADE by the authors) has also been partially characterized and contained the backbone protein, Cas7 (Csa2), Cas5a, Csa5, Cas6 and crRNAs [77]. Although not directly shown, based on the data from other type I systems, the type I-A Cascade is likely to consist of the Cas6 endoribonuclease bound to the crRNA, a backbone of six Cas7 proteins and a Cas5a/Csa5 heterodimer.

During the final step of interference, the type I Cascade containing a specific crRNA recognizes and binds the appropriate target DNA (Figure 3C). In type I-E systems, a short loop within CasA recognizes potential targets via the PAM sequence and it is proposed to bind them before the target DNA is incorporated into Cascade [78]. In addition to the PAM requirement in the target, a non-contiguous 7 nt sequence (nucleotides in position 1–5 and 7–8) within the first 8 nt of the spacer, relative to the 5' end of the spacer within the crRNA, are essential for initial target recognition and hence are termed the ‘seed sequence’ [48,79]. Cascade with bound crRNA specifically binds to negatively supercoiled target DNA [80]. The negative supercoiled topology serves as energy source for formation of an R-loop. Furthermore, Cascade induces DNA bending before Cas3 is recruited for subsequent DNA degradation (Figure 3C) [80]. Cas3 is the signature protein of type I systems and possesses ATP-dependent helicase activity as well as the ability to cleave ssDNA via an HD nuclease domain [81,82,83]. Even though the exact mechanism is not yet fully understood, it is hypothesized that once recruited, Cas3 further unwinds and cleaves the target DNA in a 5' to 3' direction, while Cascade dissociates from the target and is recycled [80,83].

#### 4.2.2. Type II Systems

A very different mechanism of CRISPR expression and crRNA maturation was discovered in the type II-A system of *Streptococcus pyogenes *[84]. An abundant short *trans*-encoded transcript is essential for processing of pre-crRNA into mature crRNA. Therefore, these RNAs are termed ***tr****ans*-**a**ctivating **cr**RNAs (tracrRNA). These tracrRNAs contain a 25 bp stretch with almost perfect complementarity to the type II CRISPR repeats within the pre-crRNA (Figure 3B). The type II repeats do not form stem-loops and the authors suggest this deficiency is overcome by pairing with the tracrRNA [84].

In addition to the tracrRNA, Cas9 (Csn1) and host RNase III are needed for the processing of pre-crRNA into mature crRNA. The requirement of RNase III is the first discovery of a non-Cas protein being an essential part of CRISPR/Cas defense machinery (Figure 3B) [84]. Interestingly, the *S. thermophilus* type II-A system provides phage resistance in *E. coli* when recombinantly expressed [59], which suggests diverse RNase III enzymes are compatible for crRNA generation. Cas9 is not only involved in crRNA processing but, along with the tracrRNA:crRNA hybrid, recognizes and degrades the target (Figure 3C) [85]. In complex with the tracrRNA:crRNA structure, Cas9 binds the target dsDNA and creates a double strand break by cleaving the complementary and non-complementary strand with its HNH- and RuvC-like domains, respectively [85]. *In vivo*, blunt cleavage of phage and plasmid dsDNA was observed at a position 3 bp from the PAM within the protospacer [53]. The PAM is important for the affinity towards the target and may have implications on the unwinding of the duplex, as cleavage of targets with mutated PAMs is only impaired in dsDNA but not ssDNA. Indeed, phages with PAM mutations also have the ability to avoid targeting by type II systems [37]. Furthermore, similar to the seed sequence in type I systems, complementarity between crRNA and target over a 13 bp stretch proximal to the PAM is required for interference [85] and hence, phages with mutations in this region of the protospacer region can evade interference [37].

#### 4.2.3. Type III Systems

In type III CRISPR/Cas systems, expression and interference are predominantly similar to those described for type I systems. However, the significant difference is that the endoribonuclease Cas6 does not bind to a repeat stem-loop but to the first bases of the repeat and that further crRNA maturation steps occur (Figure 3B) [86,87]. Indeed, not all repeats in those systems contain a strong palindrome and those associated with type III-A are predicted to be mostly non-structured [34]. In *Pyrococcus furiosus*, the binding site of Cas6 is located within nt 2–8 of the repeat and cleaves at a distal site between bases 22 and 23 resulting in crRNAs with an 8 nt repeat handle on the 5' end, the spacer sequence and 22 nt of the following repeat at the 3' end [86,87,88]. Interestingly, this RNA is not the final mature crRNA product. Cas6, unlike the type I endoribonucleases, only transfers the crRNA to the targeting complex termed CMR for type III-B where they are further processed (Figure 3C) [10,89,90]. In the type III-A system of *S. epidermidis*, a similar mechanism for processing by Cas6 was observed and the *csm2*, *csm3* and *csm5* genes were required for further 3' processing and crRNA maturation [60].

The *P. furiosus* CMR complex includes the full subset of Cmr proteins (Cmr1–6) and can contain two different species of crRNA [90], both with the 8 nt 5' end derived from the repeat and either 31 nt or 37 nt of the spacer [91]. The type III-B CMR complex from *S. solfataricus* contains an additional protein, Cmr7 and a stoichiometry of one protein of each of Cmr1–6 and 6 Cmr7 proteins [92]. Interestingly, the *P. furiosus* CMR complex has been shown to target RNA [89,90,92]. Basepairing between the last 14 nt of the crRNA and the RNA target seem to be essential for cleavage [90]. Target RNA cleavage occurs 14 nt upstream from the 3' end of the two mature crRNA species, generating two cleavage sites [90]. In contrast to type III-B, DNA is the target of type III-A systems [27], but there is not yet data about complex formation for the Csm proteins. 

The ability of CRISPR/Cas systems to target DNA raised the question of how they avoid targeting their own CRISPR arrays, which have perfect complementarity to the crRNAs they produce. For the type III-A system, this is avoided by requiring spacer:protospacer complementarity and an absence of base-pairing to the 5' handle in the crRNA [93]. This ensures that only non-self targets are licensed for degradation, but whether the same principle applies to other types remains unknown. 

## 5. Regulation of CRISPR/Cas Systems

**Figure 4:**
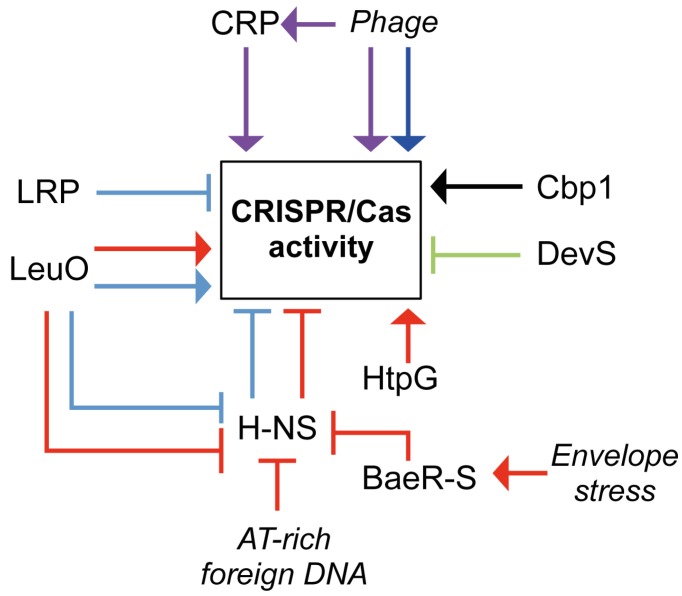
**Regulation of CRISPR/Cas activity. **Network model summarizing the regulation of CRISPR/Cas activity. Triangular and flat arrowheads indicate positive and negative effects on CRISPR/Cas activity, respectively for *E. coli* (red), *Salmonella* (cyan), *M. xanthus* (green), *Sulfolobus* (black), *T. thermophilus* (purple) and *S. thermophilus* (blue). For details see text.

As CRISPR/Cas systems function as a defense mechanism their expression might be expected to respond to invasion by extrachromosomal elements. This was recently proven in a shotgun proteomics approach in *S. thermophilus*, which showed an increase in Cas protein expression following phage infection [94]. Likewise, CRISPR and Cas expression were increased by phage infection in *Thermus thermophilus* [39]. Furthermore, CRISPR/Cas systems can be modulated in response to UV and play a role in sensitivity to DNA damage, hinting towards other possible roles besides the neutralization of invading elements [63,67]. However, to date little is known about how CRISPR/Cas systems are regulated in response to those external stimuli or during periods in which the system is not required [63,67]. Under certain conditions it could be viewed as favorable to down-regulate CRISPR/Cas activity when beneficial elements are available. However, most evidence demonstrates up-regulation of CRISPR/Cas upon exposure to phage. Since CRISPR/Cas systems are not 100% effective, some beneficial elements will be acquired and when they provide a selective advantage they are likely to be maintained. An overview of the regulatory inputs is shown in Figure 4.

Most information about CRISPR/Cas regulation is available for the type I-E systems in *E. coli* and *Salmonella enterica *serovar Typhi backgrounds. The Histone-like nucleoid structuring protein (H-NS) is a global regulator involved in compacting the bacterial chromosome, via high affinity binding to AT-rich, curved DNA (reviewed by Dorman *et al*. [95]). Many promoters are located in close proximity to curved DNA. Binding of H-NS to these promoters prevents RNA polymerase (RNAP) binding, which results in gene-silencing [95]. In *E. coli*, H-NS negatively regulates *cas* gene expression, due to H-NS binding sites near the *cas* operon promoters [41,96]. It is hypothesized that H-NS will bind to invading nucleic acids when they enter the cell, due to the higher AT-content [97,98]. Sequestration of H-NS is predicted to free the *cas* and/or LeuO promoter (see below) for RNAP recognition and thus activate expression, allowing effective CRISPR/Cas-mediated defense [41,96,99].

LRP (**l**ysine-responsive **r**egulatory **p**rotein) is a negative regulator for *cas* expression in *S. typhi* [100]. LRP functions in a similar fashion to H-NS by binding to the *cas* promoter and competitively excluding binding of RNAP. This appears to be independent of H-NS. However, LRP is able to bind simultaneously alongside H-NS, indicating that the two proteins interact to generate a nucleosome structure for *cas* gene repression [100]. Interestingly, LRP does not regulate the *cas* operon in *E. coli*, suggesting that the type I-E CRISPR/Cas systems in these two closely-related organisms function differently at the regulatory level [41,100].

LeuO, a LysR-type transcriptional regulator that also responds to amino acid starvation, affects *cas* gene expression via competition with H-NS in *S.* Typhi and *E. coli* [96,100,101]. LeuO binds to a region flanking the *cas* promoter and the H-NS binding site. The binding of LeuO to the DNA competes with H-NS binding, and relieves the inhibition of *cas* expression by enabling promoter recognition by RNAP [96,100,101]. During amino acid starvation expression of LeuO is increased in response to accumulation of the small molecules guanosine 3'-diphosphate 5'-triphosphate and guanosine 3',5'-bis(diphosphate), collectively called (p)ppGpp [102]. Interestingly, accumulation of (p)ppGpp does not occur during phage lambda infection in *E. coli* [103]. In theory, it is possible that infection with other phages triggers amino acid starvation, leading to LeuO-dependent activation of the CRISPR/Cas system.

The BaeR-S two-component regulatory system is activated in response to envelope stress (reviewed by MacRitchie *et al*. [104]). Phage infection potentially causes envelope stress by accumulation of viral proteins in the membrane. Upon detection of membrane stress, the histidine sensor kinase BaeS is activated by phosphorylation and in turn activates the BaeR protein. Activated BaeR gains the ability to bind DNA, modulating gene expression [104]. In *E. coli*, the BaeR binding site is located at the H-NS binding site near the *cse1* (*casA*) promoter. Therefore, bound BaeR serves as an antagonist for H-NS binding, predicted to result in the release of the *cas* promoters for RNA polymerase recognition and *cas* expression [105,106]. 

The high-temperature protein G (HtpG) chaperone affects the CRISPR/Cas system in *E. coli* by stabilizing Cas3. If HtpG is absent, the CRISPR/Cas system is no longer able to efficiently prevent phage infection [107]. However, HtpG is not present in all bacteria containing CRISPR/Cas systems. Indeed, most archaeal strains that contain Cas3 homologues do not carry an HtpG homolog, suggesting that it is not universally required. Therefore, other bacteria may require other chaperones in order for CRISPR/Cas interference to occur [108]. 

Other systems that affect CRISPR/Cas regulation were identified in *Myxococcus xanthus*, *T. thermophilus* and Sulfolobales. In *Myxococcus*, the *dev* operon is co-transcribed with *cas *genes, and this operon is negatively auto-regulated by DevS (a Cas5 protein) [109]. *T. thermophilus* contains 12 CRISPRs, and 3 different *cas* operons, type I-E, type III-A and type III-B [10,39]. During phage infection, the *cas* genes for the type I-E and type III-A systems were up-regulated via CRP (**c**AMP **r**eceptor **p**rotein) [39,110]. When CRP is bound to cAMP, it recognizes promoters and activates gene expression. Interestingly, inactivation of *crp* did not abolish the *cas* activation, suggesting both *crp*-dependent and -independent pathways. In addition, some *cas *genes and CRISPR arrays were regulated by phage infection in a CRP-independent manner [39]. In *Sulfolobus islandicus* and *S. solfataricus*, the expression of pre-crRNA is regulated by Cbp1 (CRISPR DNA repeat binding protein), which directly binds the DNA repeats and might influence the activity of promoters and terminators present within some spacers or repeats [111,112]. In summary, CRISPR/Cas systems can be regulated at the level of transcription of the *cas* genes and CRISPR arrays and post-translational level on the Cas proteins (Figure 4). The picture of how these systems are regulated is far from complete and diverse and complex regulatory strategies exist. 

## 6. Conclusions

CRISPR/Cas systems are widespread and versatile prokaryotic defense mechanisms providing adaptive and heritable immunity to extrachromosomal elements. In recent years research performed on these systems has led to a deeper understanding of the underlying mechanistic principles. Despite this rapid increase in knowledge, many questions remain to be answered. For example, challenges in the future include elucidating why some prokaryotes carry multiple CRISPR/Cas types and how their activity is coordinated. Furthermore, the adaptation step is still poorly understood, especially with respect to the specific roles of the Cas proteins involved and how foreign DNA is selected for integration. Major progress has been made regarding structure, function and interactions of some Cas proteins and their complexes. However, for many subtype-specific proteins, functional and structural information is still lacking and their precise role in the CRISPR/Cas mechanism remains unknown. Finally, the question, how CRISPR/Cas activity is regulated and to what extend it plays a role in areas not related to defense, e.g., DNA repair, needs to be addressed. Future research on CRISPR/Cas systems will also open up vast opportunities to utilize them, such as genome engineering for the creation of more robust industrial starter strains or as a gene targeting or gene silencing mechanism similar to that used in eukaryotic cells.
